# Use of *Pinus sylvestris* L. (*Pinaceae*), *Origanum vulgare* L. (*Lamiaceae*)*,* and *Thymus vulgaris* L. (*Lamiaceae*) essential oils and their main components to enhance itraconazole activity against azole susceptible/not-susceptible *Cryptococcus neoformans* strains

**DOI:** 10.1186/s12906-018-2219-4

**Published:** 2018-05-03

**Authors:** Daniela Scalas, Narcisa Mandras, Janira Roana, Roberta Tardugno, Anna Maria Cuffini, Valeria Ghisetti, Stefania Benvenuti, Vivian Tullio

**Affiliations:** 1 Department of Public Health and Pediatrics, Microbiology Division, University of Torino, via Santena 9, 10126 Turin, Italy; 2 Department of Life Sciences, University of Modena and Reggio Emilia, via Campi 183, 41125 Modena, Italy; 3 Microbiology and Virology Laboratory, Amedeo di Savoia Hospital, corso Svizzera 164, 10149 Turin, Italy

**Keywords:** *Cryptococcus neoformans*, Essential oils, Itraconazole, Antifungal activity, Combinatorial interaction

## Abstract

**Background:**

Cryptococcal infections, besides being a problem for immunocompromised patients, are occasionally being a problem for immunocompetent patients. In addition, the lower susceptibility of this yeast to azoles is a growing problem in health care. To date, there are very few molecules with any activity towards *Cryptococcus neoformans*, leading to heightened interest in finding new alternatives or adjuvants to conventional drugs for the treatment of mycosis caused by this yeast. Since the essential oils (EOs) are considered as a potential rich source of bioactive antimicrobial compounds, we evaluated the antifungal activity of *Origanum vulgare* (oregano), *Pinus sylvestris* (pine), and *Thymus vulgaris* (thyme red) EOs, and their components (α-pinene, carvacrol, thymol) compared with fluconazole, itraconazole, and voriconazole, against *C.neoformans* clinical strains. Then, we investigated the effect of EOs and components in combination with itraconazole.

**Methods:**

EO composition was analysed by Gas chromatography-mass spectrometry (GC-MS). A broth microdilution method was used to evaluate the susceptibility of *C.neoformans* to azoles, EOs and components. Checkerboard tests, isobolograms and time-kill assays were carried out for combination studies.

**Results:**

Six *C.neoformans* isolates were susceptible to azoles, while one *C.neoformans* exhibited a reduced susceptibility to all tested azole drugs. All EOs exerted a good inhibitory activity against all *C.neoformans* strains. Pine EO was the most effective. Among components, thymol exerted the most remarkable activity. By checkerboard testing and isobolographic analysis, combinations of itraconazole with oregano, pine, or thyme EOs, and carvacrol were found to be synergistic (FICI≤0.5) against azole susceptible *C.neoformans*. Regarding the azole not susceptible *C.neoformans* strain, the synergistic effect with itraconazole was observed with thyme EO (chemotype: thymol 26.52%; carvacrol 7.85%), and carvacrol. Time-kill assays confirmed the synergistic effects of itraconazole and oregano or thyme EO against azole susceptible *C.neoformans*. Binary mixtures of itraconazole/thyme EO or carvacrol yielded additive effects on the azole not susceptible *C.neoformans*.

**Conclusions:**

Our findings highlight the potential effectiveness of thyme, oregano EOs, and carvacrol as natural and cost-effective adjuvants when used in combination with itraconazole. Identification of EOs exerting these effects could be one of the feasible ways to overcome drug resistance, reducing drug concentration and side effects.

## Background

Systemic fungal infections caused by *Cryptococcus neoformans*, an encapsulated pathogenic yeast, are a serious health concern for immunocompromised patients worldwide, and occasionally in immunocompetent subjects [1, 2]. Cryptococcal infection occurs by inhalation of dried yeast cells from avian excreta, especially from *Columba livia* pigeons, causing potentially severe pulmonary infection, followed by haematogenous spread to the central nervous system, with meningoencephalitis being the predominant clinical presentation in human HIV-infected patients [1]. The currently recommended therapy for cryptococcosis is amphotericin B (AMB), due to its high fungicidal activity in the central nervous system, usually in combination with 5-flucytosine. However, long-term treatment with AMB has certain drawbacks due to toxic side effects (i.e. nephrotoxicity and hepatotoxicity). In addition to AMB formulations, low-dose fluconazole (FLC) and itraconazole (ITC) are used as long-term maintenance therapy of cryptococcosis, whereas voriconazole (VRC) and posaconazole (POS) are used as consolidation therapy. Compared to other azoles, ITC has a lower toxicity, and a better therapeutic index, which allow using this drug even for organ transplant, and AIDS patients [3]. Despite the effectiveness of these drugs, many recent studies indicate that the widespread use of azoles, mainly FLC, is associated with the emergence of drug-resistant isolates, and related treatment failures and infection relapses, during long-time or repeated treatment [4]. To date, there are few other molecules with any activity towards *C.neoformans*, leading to heightened interest in finding new alternatives to conventional drugs for the treatment of mycosis caused by this yeast [1]. Particularly, essential oils (EOs) have emerged in recent years as potential natural and economic alternatives or as adjuvants in combination with conventional antifungal agents and continue to be of great interest [5–10]. A large number of EOs and their major components have shown to exhibit a wide range of biological properties (antibacterial, antifungal, antiviral, anti-inflammatory, anticancer) [11–16]. Moreover, as EOs are multicomponent, there is a low probability by microorganisms to develop resistance to this mixture of substances than to a single target.

Notably, *Pinus sylvestris* L. (*Pinaceae*), *Origanum vulgare* L. (*Lamiaceae*), and *Thymus vulgaris* L. (*Lamiaceae*) EOs, as well as their main components (α-pinene, carvacrol, and thymol) have long been known for their wide use in phytomedicine, thanks to their numerous beneficial properties, including antibacterial and antifungal activities [16, 17]. A smaller number of comprehensive studies have focused on interaction of these EOs and their major components with available antifungal drugs against *C.neoformans* [18]. Conversely, these EOs and main components have been widely investigated against a wide range of bacteria [19, 20], yeasts, especially *Candida spp.* [12, 13, 21], moulds [15], but to a lesser extent on *C.neoformans* [22, 23].

In this study, we evaluated the antifungal activity of *P.sylvestris* (pine), *O.vulgare* (oregano), and *T.vulgaris* (thyme red) EOs, and their main components (α-pinene, carvacrol, and thymol), in comparison with that exerted by FLC, ITC and VRC against *C.neoformans* clinical isolates from HIV-infected patients with cryptococcosis. Then, we investigated the effect of EOs and EO components in combination with ITC against *C.neoformans* isolates. Unlike FLC, ITC is a very lipophilic drug, which may enhance penetration into the yeast cell, allowing its use also in combination with other high lipophilic compounds, such as EOs.

## Methods

### Essential oils and main components

Commercial EOs of *P.sylvestris* L., *Pinaceae* (pine), and *T.vulgaris* L., *Lamiaceae* – thymol chemotype (thyme red) were purchased from Azienda Agricola Aboca (Sansepolcro, Arezzo, Italy) as steam distilled samples. *O. vulgare* L., *Lamiaceae* (oregano) EO was obtained by hydrodistillation and kindly provided by Herboris Orientis Dacor (Milan, Italy). EO main components (positive enantiomer (+) of α-pinene, carvacrol, and thymol: ≥98% purity) were purchased from Sigma-Aldrich (Milan, Italy) and used as received without any further purification. All samples were protected from light and humidity and stored at 4 °C until use.

### GC-MS analysis

All reference standards used for GC analysis were of chromatographic grade and were purchased from Sigma-Aldrich (Milan, Italy). Chromatographic grade organic solvents were from Sigma-Aldrich (Milan, Italy). Analyses were performed on a 7890A gas chromatograph (Agilent Technologies, Waldbronn, Germany), coupled with a 5975C Network mass spectrometer (Agilent Technologies). The compounds were separated on an HP-5 MS cross-linked poly-5% diphenyl-95% dimethyl polysiloxane (30 m × 0.25 mm i.d., 1.00 mm film thickness) capillary column (Agilent Technologies). The column was initially 45 °C, then increased to 100 °C at a rate of 2 °C/min then it was raised to 250 °C at a rate of 5 °C/min and finally it was held for 5 min. The injection volume was 0.1 μl, with a split ratio 1:50. Helium was used as the carrier gas at a flow rate of 0.7 ml/min. The injector temperature was set at 250 °C. MS detection was performed with electron ionization (EI) at 70 eV, operating in the full-scan acquisition mode in the *m/z* range 40–400. EOs were diluted 1:20 (*v*/v) with *n*-hexane before GC-MS analysis [5].

### GC-FID analysis

Analyses were carried out on a 7820 gas chromatograph (Agilent Technologies), with flame ionization detector (FID). The compounds were separated on a HP-5 cross-linked poly-5% diphenyl-95% dimethyl polysiloxane (25 m × 0.2 mm i.d., 0.25 mm film thickness) capillary column (Agilent Technologies). The injection volume and the temperature program were the same as described above. The split ratio was 1:20. Helium was used at a flow rate of 1 ml/min. The injector and detector temperatures were set at 250 °C and 300 °C, respectively. EOs and reference standards were diluted 1:20 (*v*/v) with *n*-hexane before GC-FID analysis [5].

### Qualitative and quantitative analysis

The compounds were identified by the comparison of their linear retention indices (LRI) relative to C_8_-C_40_ *n*-alkanes (Sigma-Aldrich, Milan-Italy) under the above-mentioned conditions with those provided in the literature [24]. Furthermore, the identification of the several constituents was carried out by the comparison of their mass spectra with those recorded in the National Institute of Standards and Technology (NIST version 2.0d, 2005) and, when necessary, identification was carried out by co-injection of available reference compounds. The relative percentage amounts of individual components were expressed as the percentage peak area relative to the total composition of the EO obtained by the GC-FID analysis. Quantitative data were acquired as the mean of triplicate analyses for each sample.

### Essential oils and their components stock solutions

Stock solutions of each EO and its components were prepared in ethanol (1:2.5) and diluted (1:20) to obtain a final concentration of 2% (*v*/v) in RPMI-1640 medium without sodium bicarbonate and with L-glutamine (Sigma-Aldrich), buffered to pH 7.0 with 0.165 M morpholinepropanesulfonic acid (MOPS) (Sigma-Aldrich) and supplemented with 0.2% glucose [15]. Tween 80 (Sigma-Aldrich) (final concentration 0.001%, v/v) was used to enhance EO solubility, with no inhibitory effect on yeast growth.

### Antifungal drugs

FLC, ITC, and VRC powders (≥ 98% purity by HPLC) were purchased from Sigma-Aldrich (n° F8929, I6657, and PZ0005, respectively). FLC stock solutions were made up in sterile distilled water, while ITC and VRC stock solutions were made up in 100% dimethylsulfoxide (Sigma-Aldrich), and stored at − 20 °C until use.

### Yeast isolates

Seven *C. neoformans* sensu *lato* clinical isolates from HIV-infected patients with cryptococcosis, admitted to Amedeo di Savoia Hospital (Turin, Italy) between January 2013 and December 2014, were tested. Yeast isolates were identified by the API ID32C identification systems (BioMérieux, Rome, Italy). Then, they were stored at − 80 °C in Microbanks™ (Pro-Lab Diagnostics, Neston, UK), and sub-cultured at least twice on Sabouraud dextrose agar (SDA, Oxoid, Milan, Italy) at 35 °C for 72 h before testing.

### Inoculum preparation

A starting inoculum of yeast cells was prepared by inoculating three or four colonies grown on SDA agar plates in sterile saline and adjusting the yeast suspension to a 0.5 McFarland turbidity standard, corresponding to ≈ 5 × 10^6^ cells/ml. The starting inoculum was diluted in RPMI 1640 (Sigma-Aldrich) broth medium to yield a final inoculum of 0.5–2.5 × 10^3^ colony forming unit/ml (CFU/ml). The inoculum size was checked by plating serial dilutions on SDA and determining the colony counts in triplicate after incubation at 35 °C for up to 72 h [12].

### Antifungal susceptibility testing

*C. neoformans* isolates were tested for in vitro susceptibility to FLC, ITC, and VRC by broth microdilution (BM) method, according to Clinical and Laboratory Standards Institute (CLSI) M27-A3 (2008) [25]. As guidelines are not available for EO susceptibility testing, the antifungal activity of EOs and EO major components was assessed following the CLSI BM assay, with some modifications [12, 15]. Minimum inhibitory concentration (MIC) determination was performed in RPMI-1640 medium with L-glutamine without sodium bicarbonate (0.2% glucose) (Sigma-Aldrich), buffered to pH 7.0 with 0.165 M MOPS (Sigma-Aldrich), using 96-well microtiter plates (Sarstedt, Milan, Italy).

A volume of 100 μl of the inoculum suspension was transferred into microtiter plates containing 100 μl of two-fold serial dilutions of FLC (range 0.06–128 μg/ml), ITC (range 0.0078–2 μg/ml), VRC (range 0.0078–32 μg/ml), EOs (range 0.07–10 mg/ml) and EO components (range 0.02–10 mg/ml), respectively. RPMI 1640 medium alone was used as growth control. The microtiter plates were incubated at 35 °C for 72 h. MICs of azoles were read as the lowest drug concentration that produced ≥50% growth inhibition compared to the growth control. EO and EO component MICs were read as the lowest concentration at which no yeast growth was observed.

Minimum fungicidal concentrations (MFCs) were determined by spot inoculating 10 μl from wells containing either azoles or EO/EO component concentrations with inhibition of yeast growth onto SDA plates, which were incubated at 35 °C for 72 h. MFC was defined as the lowest concentration resulting in no growth on subculture, and corresponding in the death of 99.9% or more of the initial inoculum [15]. Due to the lack of clinical breakpoints, the recently published epidemiological cutoff values (ECVs) were used in defining *C. neoformans* resistance profile to FLC (ECV, 16 μg/ml), ITC (ECV, 1 μg/ml), and VRC (ECV, 0.25 μg/ml), respectively [26].

### Checkerboard assays and assessment of FIC index

Combinatorial effects between ITC and EOs/EO main components were evaluated by the checkerboard broth microdilution assay. Based on the antifungal susceptibility testing results, one azole susceptible (ADS 37) and one not-susceptible (ADS 006) *C.neoformans* isolates were selected to assess drug synergism. Therefore, a two-dimensional checkerboard with serial twofold dilutions of each compound, ranging from several dilutions below the MIC to 2 × MIC, was set up. Binary combinations were mixed within a 96-well microtiter plate. Then, yeast cell suspensions were prepared to yield final inoculum of ~ 1.5 × 10^3^ CFU/ml, and were added to each well containing binary mixtures of either ITC/EOs or ITC/EO main compounds. Each strain was tested in duplicate. Plates were incubated by shaking (150 rpm) at 35 °C for 72 h, and, afterwards, they were read visually.

The results were analysed using the fractional inhibitory concentration index (FICI). FICI values were determined by considering all first clear well in each row of the microplate containing combinations of ITC and EO/EO main components with no visible yeast growth. FICI was calculated as follows: FICI=FICa+FICb = MICa in combination/MICa tested alone+MICb in combination/MICb tested alone; where MICa and MICb are the MICs of ITC and of each tested EO used alone against *C. neoformans*, respectively. Synergy and antagonism were defined by FICI values of ≤0.5 and > 4, respectively. A FICI value between 0.5 and 1.0 was considered as additive, while a value between 1.0 and 4.0 was considered as indifferent [27].

### Isobolograms

Results of the checkerboard assays were represented graphically by isobolograms. Corresponding FIC values along the growth-no growth interface were calculated and reported by plotting FIC values of ITC along the ordinate, and FIC values of EOs/EO components along the abscissa. The straight line that joins the intercept points, corresponding to combined effects equal to the sum of the individual compounds, is the line of additivity (FICI = 1). Below this line we find the area of additive (0.5 < FICI< 1) and synergistic (FICI≤0.5) effects, respectively. FIC index values above of the straight line were interpreted as indifferent (1 < FICI< 4) or antagonistic (FICI> 4) interactions [27].

### Time-kill studies

Time–kill assays were performed with binary synergistic mixtures of either ITC/EOs or ITC/EO main components according to the results of the checkerboard assays against *C. neoformans* ADS 37 and *C. neoformans* ADS 006 strains. To examine the rate of killing of each combination, oregano, pine and thyme red EOs, and carvacrol were tested either alone or in combination with ITC at sub-MIC levels. For each tested strain, yeast cell suspension was prepared in sterile saline solution (0.85% NaCl) and turbidity was adjusted with 0.5 McFarland standard (approximately 10^6^ CFU/ml). Then, yeast suspension was diluted in RPMI medium to yield a final inoculum concentration of ~ 5 × 10^3^ CFU/ml. Test tubes containing binary combinations or compounds alone, were inoculated with the yeast suspension. Untreated yeast cells were used as growth control. For each strain tested, assays were performed in duplicate.

At predetermined time points (0, 2, 6, 24, 48 and 72 h) of incubation by shaking (150 rpm) at 35 °C, aliquots of 500 μl were removed and serially ten-fold diluted in sterile water for colony counting. A 100 μl sample from each dilution was spread onto SDA plates. After 72 h incubation at 35 °C the mean number of CFU/ml was determined, and viable counts were plotted against time on a log_10_ scale. Reduction in viable counts ≥2 log_10_ after 72 h incubation in comparison with the cell count of the most active single substance was interpreted as synergy. Additivity was defined as a 1–2 log_10_ decrease in viable counts, whereas antagonism was defined as a > 1 log_10_ increase in viable counts [27].

### Statistical analysis

All experiments were performed in duplicate and repeated at least twice. The time-kill data were analysed with one-way ANOVA followed by Bonferroni test (GraphPadPrism7, San Diego, CA, USA). The threshold for statistical significance was set at a *p* value of < 0.05.

## Results

### Composition of the EOs

The phytochemical composition of oregano, pine and thyme red EOs used for this study was determined by GC-MS and results were reported in Table 1. Pine EO was found to be rich in α-pinene (55.76%) with lower percentages of β-pinene (9.034%) and of limonene (9.74%). In thyme red EO the two major components were thymol and its precursor p-cymene (26.52%, and 16.26%, respectively), followed by a high content of limonene (13.20%), α-pinene (11.50%), carvacrol (7.85%), and γ-terpinene (4.02%). Oregano EO was found to be rich in carvacrol (62.61%), its precursor p-cymene (12.36%), and in γ-terpinene (7.60%) (Table 1). Particularly, the phytochemical composition of oregano EO proved to be highly rich in phenolic monoterpenes, in accordance with data reported in European Pharmacopoeia 9th Ed., and only minor differences were observed [28].

**Table 1:** Chemical composition of tested essential oils as determined by GC-MS analysis

Number	Component^a^	LRI^b^	*Origanum vulgare* %	*Pinus sylvestris* %	*Thymus vulgaris* %
1	α-Thujene	926	0.34	0.02	0.27
2	α-Pinene	933	0.95	55.76	11.50
3	Camphene	947	0.17	1.79	1.10
4	Sabinene	973	–	0.13	–
5	β-Pinene	979	0.60	9.034	0.77
6	β-Myrcene	992	2.34	3.41	1.30
7	α-Phellandrene	1004	0.17	0.19	0.10
8	δ-3-Carene	1009	–	6.96	1.30
9	α-Terpinene	1017	1.08	0.47	0.49
10	*p*-Cymene	1025	12.36	2.01	16.26
11	Limonene	1029	0.54	9.74	13.20
12	1,8-Cineole	1033	–	–	1.29
13	*trans*-Ocimene	1048	–	0.03	0.03
14	γ –Terpinene	1059	7.60	0.10	4.02
15	Terpinolene	1088	–	0.81	0.40
16	*cis*-Sabinene hydrate	1098	–	0.06	0.13
17	*trans-*Pinocarveol	1140	–	0.12	–
18	Camphor	1144	–	0.07	1.37
19	Borneol	1166	–	0.12	0.69
20	Terpinen-4-ol	1179	0.86	0.21	0.63
21	*p*-Cymen-8-ol	1186	–	0.25	0.04
22	α-Terpineol	1192	–	0.87	1.22
23	Verbenone	1210	–	0.07	–
24	Bornyl-acetate	1289	–	0.95	–
25	Thymol	1295	5.12	–	26.52
26	Carvacrol	1305	62.61	–	7.85
27	α-Cububene	1355	–	0.11	–
28	α-Copaene	1381	–	0.14	–
29	β-Caryophyllene	1425	0.89	0.94	1.34
30	α-Humulene	1460	–	0.11	–
31	γ –Muurolene	1481	–	0.06	–
32	Caryophyllene oxide	1593	1.01	–	–

^a^Compounds are listed in order of elution

^b^Linear retention index (LRI) calculated on HP-5 column

### Antifungal susceptibility testing

Based on susceptibility testing results, six *C. neoformans* isolates were found to be susceptible to FLC (MIC range = 0.25–4 μg/ml), ITC (MIC range = 0.25–0.5 μg/ml), and VRC (MIC range = 0.015–0.125 μg/ml), while one *C. neoformans* was found to exhibit a reduced susceptibility to all tested azole drugs, as MIC values were above the reference ECVs (Table 2). Amongst EOs, pine EO exhibited a high inhibitory activity on the growth of all *C. neoformans* azole susceptible isolates, with low MIC values ranging from 0.07 to 0.27 mg/ml (Table 2). As reported in Table 2, oregano and thyme red EOs were fairly effective in inhibiting *C. neoformans* growth, displaying MICs ranging from 0.3 to 0.6 and from 0.56 to 1.12 mg/ml, respectively. Finally, all EOs displayed a good antifungal activity against the *C. neoformans* isolate with low susceptibility to azoles, and the rank order of the most effective EOs decreased as follows: oregano>pine>thyme EO (Table 2).

**Table 2:** In vitro antifungal activity of azoles and essential oils against *Cryptococcus neoformans* azole-susceptible and not-susceptible clinical isolates

Yeast strains	Antifungal drugs (μg/ml)			Essential oils and main components (mg/ml)					
	MIC/MFC			MIC/MFC					
	FLC	VRC	ITC	*Origanum vulgare*	*Pinus sylvestris*	*Thymus vulgaris*	(+)-α-pinene	Carvacrol	Thymol
ADS 16	0.25/4	0.015/0.06	0.5/0.5	0.3/0.3	0.27/0.54	0.56/1.12	1.07/1.07	0.6/1.2	0.02/0.04
ADS 37	0.25/4	0.06/0.25	0.5/0.5	0.3/0.6	0.14/0.14	0.56/0.56	0.54/0.54	0.6/1.2	0.04/0.04
ADS 48	0.5/8	0.015/0.06	0.25/0.5	0.3/0.3	0.14/0.14	0.56/0.56	0.54/0.54	0.6/0.6	0.04/0.08
ADS 57	4/32	0.125/0.5	0.5/0.5	0.3/0.3	0.07/0.14	1.12/1.12	0.54/0.54	0.6/1.2	0.04/0.04
ADS 108	0.25/4	0.015/0.06	0.25/0.5	0.6/0.6	0.07/0.07	0.56/1.12	0.54/0.54	0.6/0.6	0.04/0.04
ADS 109	4/32	0.015/0.06	0.25/0.5	0.6/0.6	0.14/0.27	1.12/1.12	0.54/1.07	0.6/1.2	0.04/0.08
ADS 006	> 128/> 128	> 32/> 32	2/> 2	0.3/1.2	0.54/1.1	1.12/2.24	1.07/1.07	0.6/0.6	0.08/0.16

FLC: fluconazole; VRC: voriconazole; ITC: itraconazole

Epidemiological cut-off values (ECVs) for each antifungal drug: FLC, 16 μg/ml; VRC, 0.25 μg/ml; ITC, 1 μg/ml [26]

Among main components, thymol was found to be the most active pure compound, displaying the lowest MIC values (range 0.02–0.08 mg/ml) towards all *C. neoformans* isolates, irrespective of their susceptibility profile to azoles. Likewise, carvacrol and α-pinene were found to be particularly effective in inhibiting the growth of all *C. neoformans* isolates (Table 2). Conversely, terpinen-4-ol displayed moderate antifungal activity, whereas γ-terpinene exhibited weak activity against *C. neoformans* (data not shown). Furthermore, MFC results were generally equivalent or one more concentration above the MICs, indicating that the selected EOs and main components were mainly fungicidal (Table 2).

### Checkerboard assays, assessment of FIC index and isobolograms

**Fig. 1:**
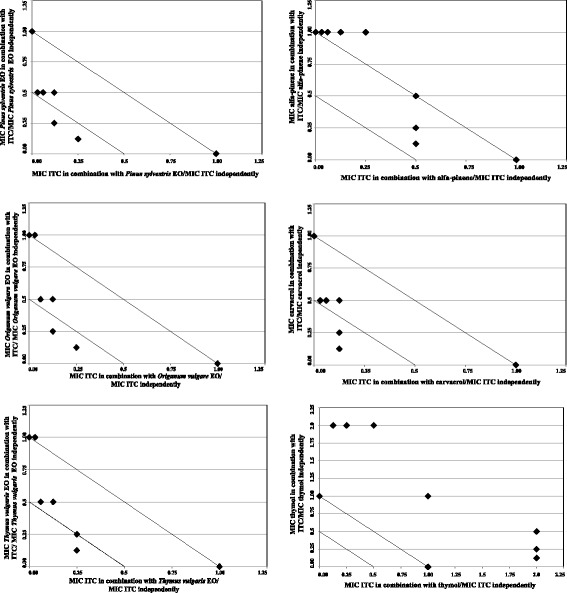
Isobologram plots of ITC and EOs/EO main components against *C.neoformans* ADS 37 (azole-susceptible strain). Points along the isobolograms represent the growth no-growth interface: fractional inhibitory concentration (FIC) values of ITC are plotted on *x*-axis, and FIC values of EOs/EO main components are plotted on *y*-axis

**Fig. 2:**
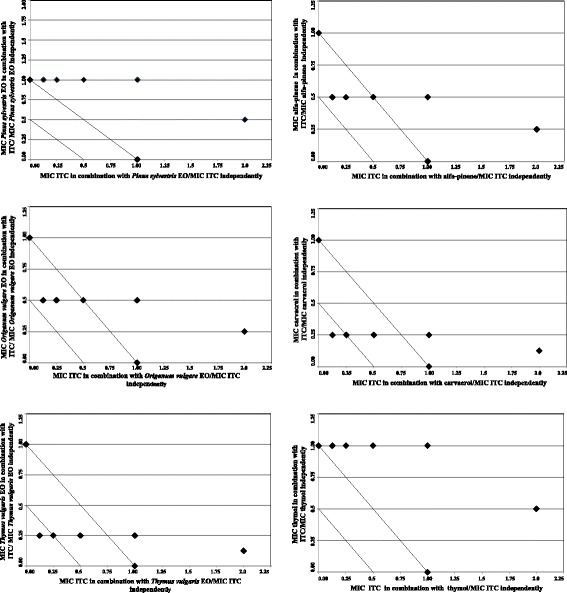
Isobologram plots of ITC and EOs/EO main components against *C.neoformans* ADS 006 (azole not-susceptible strain). Points along the isobolograms represent the growth no-growth interface: fractional inhibitory concentration (FIC) values of ITC are plotted on *x*-axis, and FIC values of EOs/EO main components are plotted on *y*-axis

The best combinations (i.e. those giving the lowest FICI values) of oregano, pine and thyme red EOs and their major components with ITC, against azole-susceptible and not-susceptible *C. neoformans* isolates were evaluated and reported in Table 3. Besides, the association profile was also exploited from all the FIC values obtained along the growth no-growth interface of each combination (Figs. 1, 2). According to the FIC indexes reported for the azole-susceptible strain, a predominantly synergistic profile (FICI≤0.5) was detected, with a consequent decrease of the MICs. In addition, the isobologram profiles were concave, confirming the presence of synergistic associations between ITC and pine, oregano, thyme EOs as well as between ITC and carvacrol (Fig. 1). Notably, when synergy was not observed, no antagonism was reported for any of the combinations. In fact, binary combinations of ITC/α-pinene and ITC/thymol resulted in either additive (FICI = 0.625) or indifferent (FICI = 2) interactions (Table 3), as also clearly depicted by the corresponding isobologram plots reported in Fig. 1.

**Table 3:** Fractional inhibitory concentration index (FICI) of essential oils *plus* itraconazole

Antifungal agents		*C. neoformans* ADS 37 (azole susceptible strain)	*C. neoformans* ADS 006 (azole not-susceptible strain)
ITC	MIC (μg/ml) alone	0.5	2
*Origanum vulgare*	MIC (mg/ml) alone	0.3	0.3
	FIC of oregano oil	0.125	0.5
	FIC of ITC	0.25	0.125
	FICI	0.375	0.625
	Interpretation	SYN	ADD
*Pinus sylvestris*	MIC (mg/ml) alone	0.14	0.54
	FIC of pine oil	0.25	1
	FIC of ITC	0.125	0.125
	FICI	0.375	1.125
	Interpretation	SYN	IND
*Thymus vulgaris*	MIC (mg/ml) alone	0.56	1.12
	FIC of thyme red oil	0.125	0.25
	FIC of ITC	0.25	0.125
	FICI	0.375	0.375
	Interpretation	SYN	SYN
(+)-α-pinene	MIC (mg/ml) alone	0.54	1.07
	FIC of α-pinene	0.125	0.5
	FIC of ITC	0.5	0.125
	FICI	0.625	0.625
	Interpretation	ADD	ADD
Carvacrol	MIC (mg/ml) alone	0.6	0.6
	FIC of carvacrol	0.125	0.25
	FIC of ITC	0.125	0.125
	FICI	0.25	0.375
	Interpretation	SYN	SYN
Thymol	MIC (mg/ml) alone	0.04	0.08
	FIC of thymol	1	1
	FIC of ITC	1	0.125
	FICI	2	1.125
	Interpretation	IND	IND

FIC of ITC = MIC of ITC in combination with EO/MIC of ITC alone. FIC of EO = MIC of EO in combination with ITC/MIC of EO alone

FICI (FIC Index) = FIC of ITC + FIC of EO. IND: indifferent; ADD: additive; SYN: synergy

Conversely, as regards the *C. neoformans* displaying low susceptibility to azoles, the synergistic effect with ITC was only obtained with binary mixtures of ITC with our thyme EO chemotype (thymol 26.52%; carvacrol 7.85%), and carvacrol, respectively (FICI = 0.375, Table 3). Oregano EO and α-pinene were found to exert additive effects (FICI = 0.625) when used in combination with ITC, whereas pine EO and thymol displayed indifferent interactions (FICI = 1.125) in association with ITC (Table 3). The isobolographic analysis of FIC values confirmed the synergistic effects of ITC/thyme EO and ITC/carvacrol binary mixtures against the azole not-susceptible *C. neoformans* strain, as in both cases the plotted isobologram points move towards the origin of the axes (Fig. 2).

### Time-kill studies

**Fig. 3:**
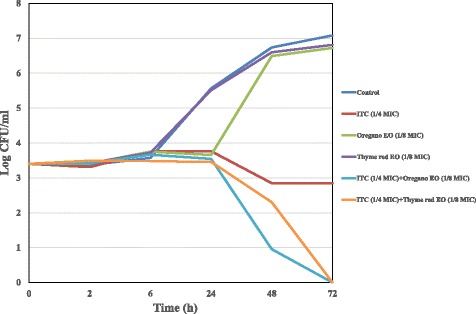
Time-kill curve of ITC, oregano and thyme red EOs alone and in combination against *C.neoformans* ADS 37 (azole-susceptible strain)

**Fig. 4:**
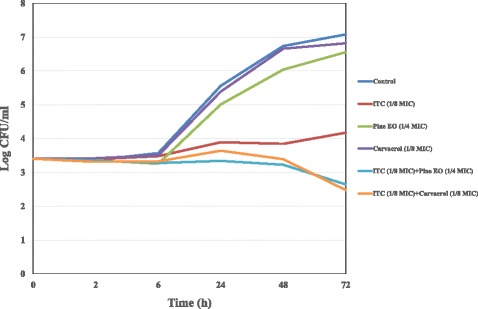
Time-kill curve of ITC, pine EO and carvacrol alone and in combination against *C.neoformans* ADS 37 (azole-susceptible strain)

**Fig. 5:**
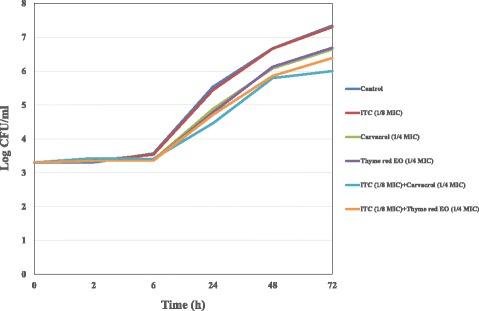
Time-kill curve of ITC, thyme red EO and carvacrol alone and in combination against *C.neoformans* ADS 006 (azole not-susceptible strain)

The death kinetics of synergistic mixtures were also investigated in time kill studies and results were reported in Figs. 3, 4 and 5. ITC at sub-MIC concentration (1/4 MIC) in combination with oregano and thyme red EOs (1/8 MIC) was found to be highly effective against *C. neoformans* azole susceptible strain, since a 2.84 log_10_ decrease in viable counts in comparison to ITC alone was detected after a 72 h time lapse (Fig. 3). Time-kill results with binary combinations of ITC (1/8 MIC) *plus* pine EO (1/4 MIC) and ITC (1/8 MIC) *plus* carvacrol (1/8 MIC) yielded a decrease of 1.53 and 1.70 log_10_ in CFU/ml in comparison to ITC alone, displaying additive effects towards *C. neoformans* ADS 37 isolate (Fig. 4). Likewise, synergistic binary mixtures of ITC (1/8 MIC) with thyme red EO (1/4 MIC) and carvacrol (1/4 MIC) produced additive effects (0.92 and 1.3 log_10_ decrease in CFU/ml) on azole not-susceptible *C. neoformans* strain by time-kill assay (Fig. 5).

## Discussion

Over the last decade, cryptococcosis has increased and has become one of the major fungal diseases of medical importance in both immunocompromised and immunocompetent individuals. Unfortunately, treatment failures, due to the emergence of yeast strains resistant to azole drugs, and mortality remain high [1]. Currently, EOs are considered as a potential rich source of bioactive antimicrobial compounds to improve antifungal treatment. Moreover, synergy research is actively studying efficacy of EOs and their main components in combination with already existing drugs, so that drug dosages and treatment-related adverse side effects can be significantly reduced, preventing or delaying the development of drug resistant strains [6–10, 18]. To date, few studies have evaluated the combinatorial effects of EOs with azoles against *C. neoformans* using in vitro synergy [18]. Therefore, in the present study we tested three EOs from Lamiaceae (*O. vulgare* and *T. vulgaris*) and Pinaceae (*P. sylvestris*) plant families, some of the best inhibitors of fungal pathogens, and single main components (α-pinene, carvacrol and thymol), against *C.neoformans* clinical strains from HIV-infected patients with cryptococcosis with a different pattern of susceptibility to azoles (FLC, ITC, and VRC). Moreover, we evaluated whether these natural compounds may enhance the activity of ITC against azole susceptible and not-susceptible *C.neoformans* isolates.

Based on antifungal susceptibility testing, pine EO displayed the highest inhibitory activity (MIC = 0.07–0.27 mg/ml) on six *C. neoformans* isolates susceptible to azole drugs (FLC, ITC, and VRC), whereas oregano EO was found to be the most effective against the azole not-susceptible isolate (MIC = 0.3 mg/ml; Table 2). As previously seen with *C. albicans* by Khan et al. [21], high antifungal activities of oregano and thyme EOs may be ascribed to the presence of phenolic components such as carvacrol and thymol, which may determine membrane deterioration through oxidative stress and alteration of the antioxidant defence system even at low concentrations. Interestingly, other authors reported that thyme EO may induce phenotypic switching on the polysaccharide capsule of *C. neoformans*, decreasing capsule size and leading to alteration of yeast virulence [29].

According to our MIC/MFC results, thymol, the main component of thyme red EO, was the most effective pure compound, exhibiting yeast growth inhibition stronger than thyme EO alone against all *C. neoformans* isolates at low MIC values ranging from 0.02 to 0.08 mg/ml (Table 2). Thymol was also more effective than carvacrol and α-pinene, the main bioactive components of oregano and pine EOs, respectively. In fact, α-pinene and carvacrol showed a good antifungal activity against all *C. neoformans* isolates, though at higher MIC concentrations (Table 2). These findings on α-pinene are consistent with previous studies and may be related to significant inhibition of *C. neoformans* phospholipase and esterase activities caused by both α- and ß positive enantiomers of pinene [30]. On the contrary, MIC results of carvacrol and thymol differ to some extent from those previously reported by other authors [22, 23, 31, 32]. However, we would like to highlight that the methodological approaches for MIC determination used in these studies were clearly different, rather not standard, and therefore susceptibility results were difficult to compare.

This study showed, for the first time, synergistic and additive effects of combinations of pine, oregano, and thyme red EOs with ITC against both azole susceptible and not-susceptible *C. neoformans* clinical isolates. According to FICI results and isobolographic analysis, pine, oregano, and thyme red EOs in combination with ITC showed a synergistic action against azole susceptible yeast strain. Among main components, carvacrol showed a synergistic profile when combined with ITC, while ITC/α-pinene and ITC/thymol combinations resulted in either additive or indifferent interactions (Table 3). In this study binary mixtures of ITC with our thyme EO chemotype (thymol 26.52%; carvacrol 7.85%), and carvacrol were found to be synergistic against azole not-susceptible *C. neoformans* strain. On the contrary, oregano EO and α-pinene were found to exert additive effects when used in combination with ITC, whereas pine EO and thymol displayed indifferent interactions in association with ITC. Antagonism was never observed.

Finally, the time-kill method revealed a more detailed insight into the antifungal activities of the synergistic combined mixtures. In agreement with checkerboard data, ITC at sub-MIC concentration (1/4 MIC) in combination with oregano and thyme red EOs (1/8 MIC) confirmed to be highly effective against *C. neoformans* azole susceptible strain (Fig. 3). Particularly, these binary combinations produced the same antifungal effects obtained when testing the singular drug at MIC level by time-kill assay (data not shown). The synergistic combination of ITC with our tested EO chemotypes (oregano and thyme red) might be explained by the EOs promoting the effects of antifungal drugs, mainly on the cell wall, plasma membrane and other membrane structures of *C. neoformans*. In addition, EO damage on yeast cell wall and membrane may facilitate ITC entry into the cell, probably leading to a greater effect on ergosterol biosynthesis inhibition and adding to *C. neoformans* membrane destruction. Overall, this issue might also turn the fungistatic action of ITC into a fungicidal action [7, 9].

Conversely, time-kill results with binary combinations of ITC (1/8 MIC) *plus* pine EO (1/4 MIC) and ITC (1/8 MIC) *plus* carvacrol (1/8 MIC) were not really consistent with those from checkerboard assays, displaying additive effects towards *C. neoformans* ADS 37 isolate (Fig. 4). Likewise, synergistic binary mixtures of ITC (1/8 MIC) with thyme red EO (1/4 MIC) and carvacrol (1/4 MIC) yielded additive interactions on azole not-susceptible *C. neoformans* strain (Fig. 5). Despite these apparently contradictory results between checkerboard and time-kill assays, these differences may be ascribed to the different methods used, as time-kill assay records a fungicidal effect, whereas the checkerboard titration reveals at least inhibition of fungal growth [27]. According to literature data, an indifferent effect was detected against both *C. albicans* and *C. neoformans* strains when α- and ß- pinene positive enantiomers of pinene were combined with AMB [30]. Moreover, other authors have found that thymol may exert synergistic interactions with AMB, FLC and ITC towards *C. albicans*, whereas it may display either synergistic or additive, or indifferent activities when combined with AMB, ITC, and FLC against *C. neoformans* strains, respectively [18].

Terpenoids/monoterpenes are the main constituents of plant-derived EOs, frequently used in folk medicines, pharmaceutical industry and cosmetics. Among monoterpenes tested in our study, both carvacrol and thymol have been classified as GRAS (generally recognized as safe) for human consumption at low concentrations, as they do not exhibit systemic toxicity. Furthemore, their use in food has been also approved by European Parliament and Council, making them a potential option for developing anti-cryptococcal drugs [31]. In addition, α, and β pinene are generally considered as safe at low concentrations and are commonly used to produce balsamic candies and fumigations [30]. Notably, thymol is also widely used in dental practice, and has shown to interact positively with the GABA(A) receptor in mouse cortical neurons [33]. Recently, some authors demonstrated through cytotoxicity assays and keratinocyte- *Cryptococcus spp*. co-culture infection models that thymol and carvacrol were efficient in terms of human safety, suggesting that these pure compounds can be further exploited as cost-effective and non-toxic anti-cryptococcal drugs [31]. Nevertheless, in human organism, other monoterpenes (e.g., pulegone, menthofuran, camphor, and limonene) have been reported to exhibit toxic effects in various organs, mostly in liver [34]. Therefore, it is always advisable that EOs should be used carefully, as they may be toxic and show adverse effects on humans when overdosed [35].

## Conclusions

Synergistic and additive EO/azole drug combinations may be potential strategies to counteract antifungal resistance, since use of multiple compounds may disrupt several fungal functions and thus minimize selection of resistant fungal strains. In this context, our findings represents an original proof-of-concept, as they highlight the potential effectiveness of oregano, thyme red EOs and carvacrol as natural and cost-effective adjuvants, at low doses (i.e., < 0.1 mg/ml) [31], when used in combination with ITC for cryptococcosis treatment.

Further in-depth studies of the modes of action for synergistic combinations, as well as in vivo preclinical models are encouraged to predict the translational use of these data for clinical settings.

## Abbreviations

AMB: Amphotericin B
BM method: Broth microdilution method
CFU: Colony forming unit
CLSI: Clinical and Laboratory Standards Institute
ECVs: Epidemiological cutoff values
EO/EOs: Essential oil/Essential oils
FICI: Fractional inhibitory concentration index
FLC: Fluconazole
GC-FID: Gas-chromatography-flame ionization detector
GC-MS: Gas-chromatography-mass spectrometry
ITC: Itraconazole
MFC: Minimum fungicidal concentration
POS: Posaconazole
SDA: Sabouraud dextrose agar
VRC: Voriconazole
